# Antimicrobial use in cattle and poultry production on occurrence of multidrug resistant *Escherichia coli*. A systematic review with focus on sub-Saharan Africa

**DOI:** 10.3389/fvets.2022.1000457

**Published:** 2022-10-24

**Authors:** Rogers Azabo, Frankwell Dulle, Stephen E. Mshana, Mecky Matee, Sharadhuli Kimera

**Affiliations:** ^1^Department of Veterinary Microbiology, Parasitology and Biotechnology, College of Veterinary Medicine and Biomedical Sciences, Sokoine University of Agriculture, Morogoro, Tanzania; ^2^National Livestock Resources Research Institute, Kampala, Uganda; ^3^Southern African Centre for Infectious Disease Surveillance (SACIDS) Foundation for One Health Sokoine University of Agriculture, Morogoro, Tanzania; ^4^Department of Knowledge Management, Sokoine National Agricultural Library, Sokoine University of Agriculture, Morogoro, Tanzania; ^5^Department of Microbiology and Immunology, Weill Bugando School of Medicine, Catholic University of Health and Allied Sciences, Mwanza, Tanzania; ^6^Department of Microbiology and Immunology, School of Medicine, Muhimbili University of Health and Allied Sciences, Dar es Salaam, Tanzania; ^7^Department of Veterinary Public Health, College of Veterinary Medicine and Biomedical Sciences, Sokoine University of Agriculture, Morogoro, Tanzania

**Keywords:** antimicrobial use, cattle, poultry, quality, quantity, sub-Saharan Africa

## Abstract

Antimicrobial use in livestock production has been linked to antimicrobial resistance (AMR) worldwide; however, optimization of their use has been considered an important strategy in dealing with it. The aims of this study were as follows: (a) to assess the literature on antimicrobial usage (practices, frequency, class, type) in cattle and poultry production with regard to resistance in *Escherichia coli* (*E. coli*) including multidrug resistance (MDR) (b) summarize evidence for quantitative (volumes of active antimicrobial ingredients) and quality (identify and quantify active ingredient) and (c) to identify data gaps. Peer reviewed literature search was conducted by querying two online databases: PubMed and Google scholar from November 15, 2018 to February 2019. The inclusion criteria for eligibility were articles: published in English between 2008 and 2018, including poultry (chicken) or cattle or both, *E. coli* bacteria of choice, antimicrobial use on farms, quantitative data and quality of antimicrobial used. Microsoft Excel was used for data extraction and Rayyan software for eligibility studies. The search retrieved 1,446 probable articles including those from the reference list of significant papers, of which twenty-four articles remained on full text review with more than a third of the studies being conducted in Nigeria. Farm surveys and antimicrobial sales were identified as the main sources of data and the mean quantities of antimicrobials based on sales data were 23,234, 41,280.87, and 1,538,443 kg of the active ingredient in Nigeria, Zambia and South Africa, respectively. One study from Cameroon determined the quantities of active ingredients based on dose metrics while another study still from Cameroon mentioned the quality of antimicrobials. Tetracyclines, beta-lactams/aminoglycosides and fluoroquinolones were the most common classes of antimicrobials (antibiotics) used. Our review reveals a dearth of information in Sub- Saharan Africa on the quantity and quality of veterinary drugs and yet they play a role in the overall picture of antimicrobial resistance. This finding gives an opportunity in the area of focus for future research as far as resistance and multidrug resistance are concerned in food producing animals.

## Introduction

Antimicrobial use (AMU) in livestock production, is not only for improving productivity and sustainability but also as growth enhancers (1). Its use involves different classes of antimicrobials of varying doses and their implementation methods depend on the livestock species and production system (2). Owing to the increasing demand for dietary protein intake of foods of animal origin, livestock production in developing economies has become intensive whereby AMU is inevitable (3, 4). However, there is mounting evidence over the years that the dependence of food producing animals on antimicrobials due to their indiscriminate and inappropriate usage has led to the selection, emergence, and spread of antimicrobial resistant bacterial strains in both animals and humans (5, 6). Although its magnitude is unknown, it is likely to vary depending on the type and quantity of antimicrobial used. This resistance phenomenon is of ultimate global health concern and the situation is worsened by the emergence of multiple drug resistance (MDR) in food animals. Increased levels of antimicrobial resistance (AMR) in livestock production either reduce farm productivity or increase disease treatment costs (7). Consequently, several calls have been made to optimize this usage in order to limit the growth of AMR in humans (8–10).

A previous study by O'Neill (10), predicted antimicrobial consumption in food animals to rise by 67% by 2030 globally, and nearly double in Brazil, Russia, India, China, and South Africa. This rise was probably attributed to the growth in consumer demand for livestock dietary products (eggs, meat, milk) in middle-income countries and a shift to large-scale farms where antimicrobials are used routinely (3, 11). Earlier studies by McEwen and Fedorka-Cray (12) and Moulin et al. (13) indicated that in Europe and the United States antimicrobials in livestock production represent the largest fraction (66–80%) of the total global usage.

AMU measurement in livestock production is of importance, as it addresses several issues among which include; monitoring AMU over time, setting benchmarks to promote AMU reduction, and correlating the association between AMU and AMR. However, data across studies cannot be compared due to diverse metric systems in the measurement or quantification of antimicrobials (14). This is further complicated by inadequate resources and research capacity which is typical of developing countries (8).

Although research has increased in recent years on the role of poor-quality veterinary medicine, its impact has not been incorporated into the overall picture of antimicrobial resistance by the scientific community (15). This knowledge gap in veterinary antimicrobials can be exploited in the emergence of antimicrobial resistance (16).

In the current article, we reviewed and summarized original peered-reviewed research articles on AMU in cattle and poultry production in sub-Saharan Africa. The aim of this study was to assess the literature on antimicrobial usage (practices, frequency, class, type) in cattle and poultry production with regard to resistance in *E. coli* including MDR, summarize evidence for quantitative (volumes of active antimicrobial ingredients) and quality (identify and quantify active ingredient) of antimicrobials from 2008 to 2018.

## Materials and methods

This review covers the use of antimicrobials in cattle and poultry production, with the following research question: What is the pattern of antimicrobial use in terms of classes and purpose; what methods are used to quantify antimicrobials and their quality with regard to the occurrence of resistance in *E. coli including MDR*? This systematic review was performed in accordance with the PRISMA (Preferred Reporting Items for Systematic Reviews and Meta-Analysis) guidelines (17). It was conducted in four steps: database search, evaluation of the articles, data extraction and Library formation/summary. Search criteria were defined and verified by researchers, and also modalities on how to settle disagreements before the initiation of the study.

### Data sources and search strategy

A multifaceted search was conducted by querying two online databases: PubMed and Google scholar between November 15, 2018 and February 2019 for published literature in English. Boolean operators (AND/OR) were used among keywords like antimicrobial usage, quantity, quality, livestock, poultry, chicken, cattle, dairy and beef followed by specific names of individual countries in sub-Saharan Africa for relevant articles published between 2008 and 2018. In addition, reference lists of relevant articles were searched manually for supplementary literature. This period (2008 and 2018) is justified by numerous studies conducted on antimicrobial use and resistance in cattle and poultry production. The final search string and the number of citations used in this study are shown in Supplementary Table 1.

### Eligibility article assessment/evaluation

The inclusion criteria were, studies; (i) published in English (ii) focused on quality, qualitative and quantitative data on antimicrobial use in poultry or cattle or other livestock species but poultry or cattle inclusive (iii) conducted between 2008 and 2018 in any of the 46 countries in sub-Saharan Africa, (iv) original research study (v) mentioned about *E. coli*. Citations of included articles were downloaded and stored as Comma delimited files. The files were eventually exported to Rayyan online application software for selection eligibility by two researchers (RA and FD). The researchers independently screened the relevant articles based on their titles and abstracts against the search criteria (first screening), followed by full text reading (second screening). Contentions in article selection were resolved on consensus by the researchers.

### Quality assessment

Articles were graded based on the grading approach by the GRADE Working Group (18) for human research, on full text review since we did not come across that for animal research. This approach grades an article on the basis of quality, directness, and consistency for quality of evidence. In our review, quality was given a score of two, one on evidence of statistical analysis and another on bias or design limitations. The directness score was based on whether the methods and results presented were clear and easily understood and the consistency score was on the fact that the results and conclusion presented appeared to be consistent with the methodology. When the three scoring categories are combined, each article could receive a maximum score of plus four (+4) and a minimum score of minus six (−6) (Table 1).

**Table 1:** Scoring system for generating a grade for articles on full text review based on the GRADE approach.

**Variable**	**Score**
**Quality**	
Statistical evidence	0 = no evidence
	+2 = evidence
Probability of bias and design limitations	0 = none
	−1 = some
	−2 = high
**Directness**	
Method and results clear and straight forward	−2 = not direct
	−1 = some uncertainty
	+1 = direct
Consistency-results and conclusion presented appear to be consistent with methods	−2 = important
	Inconsistence
	−1 = some
	Inconsistence
	+1 = consistent

### Data extraction and management

Data from eligible study articles were extracted and summarized onto a Microsoft^®^ Office Excel 2007 framework sheet by RA and revised independently by FD. For each article, information was documented systematically in detail of publication (country, author, year of study, study unit, sample type, study population (cattle, poultry, goat, sheep, and pigs) and antimicrobial use (AMU), Supplementary Table 2. To minimize bias, articles were carefully scrutinized during data extraction due to variations in study execution and reporting methodologies. In circumstances where information was not clear, the onus was upon the researchers to either include or exclude it on full text review or contact the author by email for clarity.

## Results

### Eligible studies

**Figure 1:**
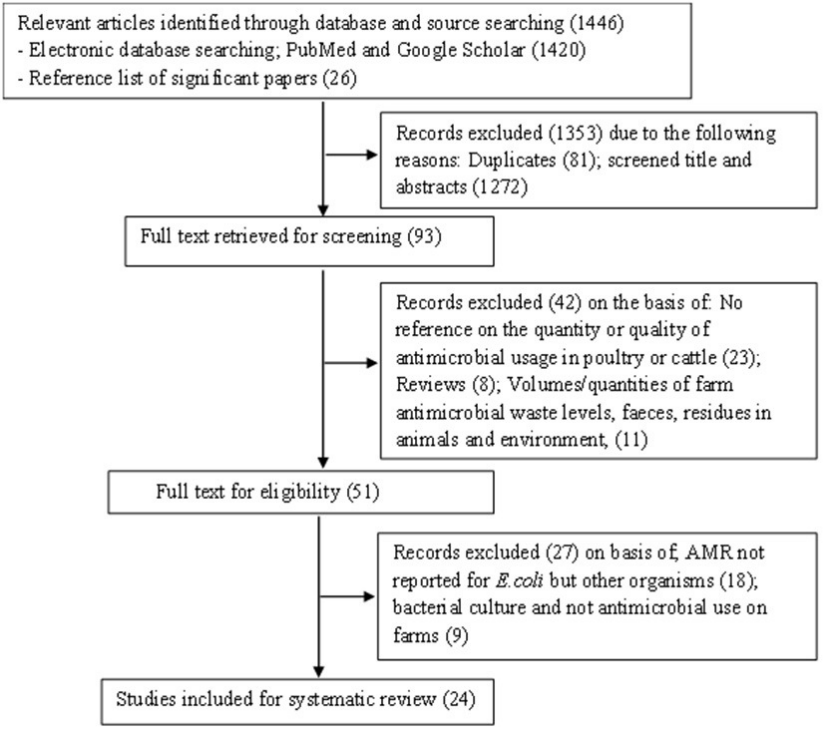
A flow diagram of the selection of eligible studies.

A total of 1,446 articles were retrieved from two online databases: PubMed and Google scholar as well as through a manual search of reference lists of relevant articles. On screening and duplicate removal, 93 articles remained for the initial title and abstract screening. Of these 51 articles were eligible for full text review based on inclusion criteria. However, twenty-seven articles were excluded with reason on full text review. In total 24 articles were included in this systematic review as shown in PRISMA flow diagram Figure 1.

### Description of the included studies and data sources

As shown in Table 2, twenty-four studies were included in the final analysis of this systematic review. Nearly a third (9/24, 38%), of the studies were conducted in Nigeria and the rest in other sub-Saharan African countries. Almost half (10/24, 42%) of the relevant articles identified were based on poultry, three on cattle (beef or dairy) and eleven on more than one animal species with either both cattle and poultry inclusive or one of them. Antimicrobial use or data (prevalence of use/antimicrobial classes, or antimicrobials sold) was mentioned in all the studies and these studies were cross-sectional in design. Two data sources were identified; farm surveys and antimicrobial sales data. Of the 24 studies, twenty collected data through farm surveys only, two compiled data from antimicrobial sales alone and two collected from both farm surveys and antimicrobial sales. Three studies estimated the quantities of antimicrobials from sales data both nationally and regionally, one study estimated the quantities based on dose metrics from farm data and only one study mentioned about the quality of antimicrobials.

**Table 2:** Summary of 24 articles on antimicrobial use (AMU) stratified by study year, country location, study design, and livestock species.

		**Number of study articles (%)**			
**Category**	**Sub-category**	**Qualitative**	**Quantitative**	**Quality**	**All types**
		**(19)**	**(4)**	**(1)**	**(24)**
Publication year	2014–2018	13 (68)	4 (100)	1 (100)	18 (75)
	2008– 2013	6 (32)	0 (0)	0 (0)	6 (25)
**Country location**					
	Nigeria	8 (42)	1 (25)	0 (0)	9 (38)
	Uganda	2 (11)	0 (0)	0 (0)	2 (8)
	Ghana	2 (11)	0 (0)	0 (0)	2 (8)
	Cameroon	1 (5)	1 (25)	1 (100)	3 (13)
	Sudan	2 (11)	0 (0)	0 (0)	2 (8)
	South Africa	0 (0)	1 (25)	0 (0)	1 (4)
	Zambia	1 (5)	1 (25)	0 (0)	2 (8)
	Tanzania	2 (11)	0 (0)	0 (0)	2 (8)
	Ethiopia	1 (5)	0 (0)	0 (0)	1 (4)
**Study designs**					
(Cross-sectional designs)	Farm surveys	18 (95)	1 (25)	1 (100)	20 (83)
	Sales data	0 (0)	2 (50)	0 (0)	2 (8)
	Farm surveys and sales data	1 (5)	1 (25)	0 (0)	2 (8)
**Animal species**					
	Poultry	9 (47)	1 (25)	1 (100)	11 (46)
	Cattle	2 (11)	1 (25)	0 (0)	3 (13)
	Combined data	8 (42)	2 (50)	0 (0)	10 (41)

### Antimicrobial use in food producing animals

Antimicrobials were used in poultry and cattle production for different purposes. They were either used for therapeutic/ prophylactic purposes or as growth enhancers. However, the highest usage was observed in poultry. Seven articles indicated that antimicrobials were mostly used for therapeutic purposes (19–25), two for prophylactic (26, 27), four for both prophylactic and therapeutic (28–31), and nine for all purposes (32–40).

Antimicrobial usage percentage on the farms varied from 67% in Nigeria to 100% in Cameroon, Nigeria and Zambia. The commonly used antimicrobial classes were tetracyclines, beta-lactams/aminoglycosides and fluoroquinolones (Table 3). Of the 24 articles, four studies reported on antimicrobial sales. One of the studies estimated a mean quantity of 1,538,443 kg over a period of 3 years (41) based on national sales in South Africa, another study reported a mean quantity of 23,234 kg over a period of 3 years (42) based on the sales in South-Western region of Nigeria while the third study reported a mean quantity of 41,280.87 kg sold over a period of 1 year in Zambia (22) and the fourth study reported on the brands of antimicrobials marketed by the drug shop outlets without specifying the volumes or quantities sold in North-Eastern Nigeria (20). However, a study by Kamini et al. (30) reported on the quantitative estimates based on dose metrics (defined daily doses) of active antimicrobial ingredients. Only one study reported quality determination using High-performance liquid chromatography (HPLC) (24).

**Table 3:** Proportions of farms using antimicrobials by country, animal type and antimicrobial class.

**References**	**Country**	**Food animal**	**% AMU**	**Antimicrobial class**	**Time period**
Adebowale et al. (27)	Nigeria	Poultry	100	Aminoglycosides, Tetracyclines, Phenicols, Sulphonamides, Nitrofurans, Macrolides, Beta-lactams, Quinolones	March–July 2011
Awogbemi et al. (38)	Nigeria	Poultry	100	Beta-lactams, Tetracyclines, Phenicols, Aminoglycosides, Macrolides, Quinolones, Sulphonamides, Nitrofurans	Not specified
Bashahun and Odoch (36)	Uganda	Poultry	96.7	Tetracycline, Sulphonamide	December 15th 2013–January 28th 2014
Boamah et al. (28)	Ghana	Poultry	98	Tetracycline, Macrolides Aminoglycosides, Polymyxins, Sulphonamides, Beta-lactams, Fluoroquinolones, Pyremethamine	June 2012–July 2013
Kamini et al. (30)	Cameroon	Poultry	100	Beta-lactams, Aminoglycosides, Polymyxins, Diaminopyrimidines, Fluoroquinoles, Macrolides, Nitrofurans, Sulphonamides, Tetracyclines	February–May 2015
Geidam et al. (20)	Nigeria	Poultry	100	Tetracycline, Aminoglycosides, Nitrofurans, Sulphonamide	February–December 2010
Oluwasile et al. (35)	Nigeria	Poultry	100	Fluoroquinolones, Nitrofurans, Tetracycline, Polymyxins, Aminoglycosides, Macrolides, Beta-lactams	March–July 2011
Alhaji and Isola (37)	Nigeria	Cattle/sheep/goats	88.5	Tetracycline, Macrolides, Beta-lactams, Aminoglycosides, Sulphonamides	November 2015–March 2016
Okpara et al. (39)	Nigeria	Poultry/goats/sheep	100	Tetracyclines, Beta-lactams, Phenicols, Fluroquinolones, Macrolides, Nitrofurans, Polymyxins, Aminoglycosides	Not specified
Eltayb et al. (33)	Sudan	Poultry/cattle/goats	95	Tetracyclines, Beta-lactams, Macrolides, Sulphonamides, Aminoglycosides, Quinolones	December 2008–April 2009
Amaechi (34)	Nigeria	Poultry and pigs	67	Tetracycline, Aminoglycosides, Macrolides	June 2011–May 2012
Mainda et al. (22)	Zambia	Cattle	–	Tetracyclines, Sulphonamides, Pencillins, Macrolides, Aminoglycosides, Polypeptides	Not specified
Caudell et al. (23)	Tanzania	Cattle/sheep/goats	74	Tetracyclines, Penicillin Aminoglycosides, Macrolides, Sulphonamides	2013–2015
Okubo et al. (40)	Uganda	Cattle/pigs/goats/chicken	100	Pencillins, Aminoglycosides, Tetracyclines, Macrolides, Fluoroquinolones, Sulphonamides	September 2016–February 2017
Donkor et al. (26)	Ghana	Cattle/goats/pigs/chicken and sheep	98	Pencillins, Aminoglycosides, Sulphonamides, Tetracyclines, Macrolides, Fluoroquinolones	July–November 2007
Tufa et al. (25)	Ethiopia	Cattle/poultry	80	Tetracycline, Sulphonamide, Penicillin, Aminoglycosides	December 2013–March 2014
Mubita et al. (19)	Zambia	Cattle	100	Tetracycline, Penicillin	Not specified
Sirdar et al. (21)	Sudan	Poultry	93	Tetracycline, Macrolides, Quinolones, Polypeptides	December 2007–January 2008
Guetiya et al. (29)	Cameroon	Poultry	80	Tetracycline, Phenicols, Aminoglycosides, Quinolones, Sulphonamides	December 2012–June 2013
Vougat Ngom et al. (24)	Cameroon	Cattle	69	Tetracyclines, Pencillins, Sulphonamides, Quinolones, Aminoglycosides	September 2011–April 2012
Nonga et al. (32)	Tanzania	Poultry	90	Tetracycline, Sulphonamides, Aminoglycosides, Quinolones, Dihydrofolate	January–February 2007
Olufemi et al. (31)	Nigeria	Cattle/poultry/sheep/goats	99.1	Beta-lactams, Macrolides Aminoglycosides, Quinolones, Nitrofurans, Tetracycline, Phenicols, Polypeptides, Polymyxins, Sulphonamides	Not specified

### Assessment of antimicrobial resistance

Seven studies reported on different antimicrobial resistance (19, 22–29, 38–40) levels within and between countries. The proportions of AMR of *E. coli* isolates ranged from 6.5% in Zambia (22) to 100% in Nigeria (38). Clinical and Laboratory Standards Institute (CLSI) (43) guidelines were used for antimicrobial susceptibility testing (AST) in most of the studies and EUCAST (European Committee on Antimicrobial Susceptibility Testing) (44) in only one study (40). Overall *E. coli* isolates were screened with varying amounts of antibiotics ranging from 6 (22) to 14 (39) across the respective studies using disk diffusion (5/6) and broth microdilution (1/6) as the main methods of AST. Susceptibility testing was frequently performed on tetracycline, gentamicin, ampicillin, chloramphenicol, ciprofloxacin, cotrimoxazole, -augmentin, trimethoprim-sulfamethoxazole nalidixic acid, amoxicillin, kanamycin, and streptomycin.

### Multidrug resistance in *Escherichia coli*

Three studies reported on multidrug resistance. The proportion of multidrug resistance (MDR) strains among *E. coli*, which is an indicator organism, is shown in Table 4. These studies defined MDR as non-susceptibility to antimicrobial agents belonging to at least three or more different antimicrobial classes (26, 39, 40). The MDR *E. coli* proportions ranged from 98.4% in Uganda (40) to 100% in Ghana (26).

**Table 4:** Percentage of MDR strains among Escherichia coli isolated from poultry and cattle.

**References**	**Sample type**	**% MDR**	**Antimicrobials of concern**	**Time period**
Donkor et al. (26)	Cattle/goats/pigs/sheep/chicken	100	Tetracycline, Ampicillin, cefuroxime, cefotaxime, chloramphenicol, gentamicin, cotrimoxazole, amikacin	July–November 2007
Okpara et al. (39)	Chicken/goats/sheep/pigs/cattle	–	Streptomycin, Sulfamethoxazole/trimethoprim, gentamicin, chloramphenicol, Kanamycin, amikacin Ciprofloxacin, ampicillin, cefotaxime, ceftazidime, trimethoprim, compound sulphonamides, Nalidixic acid tetracycline,	Not specified
Okubo et al. (40)	Cattle/goats/pigs/chicken	98.4	Ampicillin, Cefazolin, Cefotaxime, Gentamicin, Kanamycin, Tetracycline, Minocycline, Nalidixic acid, Ciprofloxacin, Colistin, Chloramphenicol, Sulfamethoxazole-trimethoprim	September 2016–February 2017

## Discussion

Information on antimicrobial use in food animals is useful for several reasons, among which include raising awareness, identification of use pattern trends over time, antimicrobial resistance data integration, and evaluation of effective measures on judicious use of antimicrobials (45). Several studies on antimicrobial use have been conducted over the past decade and in this review, most of the studies were between 2008 and 2018. The majority of 9/24 of the studies were conducted in Nigeria. This implies that the public health significance of resistance to food production animals is recognized in Nigeria by the government since it provides research funding as reflected in two studies (27, 31). Although other studies in Nigeria did not indicate the source of funding.

### Article type

We reviewed 24 articles on antimicrobial use in poultry and cattle production published in English since 2008. A number of articles (*n* = 22) reported on qualitative (proportion) usage on farms with time frames although a few did not specify. The importance of the time frame is that it simplifies the interpretation of data since usage is dependent on the observation period. Information on proportion of usage is important among other reasons; such as comparing use patterns across countries and conducting risk assessments. Interestingly, only one article from Cameroon was identified on the quality of antimicrobials (24). Although not verified, probably this reflects language bias, as it is likely that some studies were published in languages other than English, or were outside the scope of the search engine. Quality of antimicrobials is of importance as low or poor quality may play a role in infection treatment failure due to incorrect active substances. One stud estimated quantities of antimicrobials based on dose metrics (30). Quantitative data is dose dependent, and when coupled with antimicrobial resistance data may potentially help in explaining the association between antimicrobial usage and antimicrobial resistance (46). Since antimicrobial active principles/substances vary in their potency, usage of dose-based metrics results in a fairer comparison between antimicrobials. However, there is no universally accepted dose standard, as these vary by country, species, route of application, and indication (47). Even if doses are standardized, estimating the number of doses from gross amounts of active ingredients is challenging because animals (especially poultry and pigs) may increase their body size over production (48).

### Data sources

In most of the studies, farm surveys and antimicrobial sales were the two main sources of data for this review. However, farm surveys were the primary source of data, since most of these countries have not yet developed a national antimicrobial use monitoring system. Farm surveys which can either be longitudinal or cross sectional, have an advantage over antimicrobial sales in that they give detailed information on the species for which the antimicrobial is being used, the purpose for the use, dosage form, treatment duration, and production type. Unreliable antimicrobial sales figures make accurate antimicrobial use data collection difficult and labor intensive. However, when comprehensive antimicrobial sales data are used in monitoring antimicrobial use trends over time, as long as the production animal population is stable. Antimicrobial use data when collated by national surveillance systems are used in determining the impact of large-scale interventions, as performed in Norway (49).

### Antimicrobial use

Antimicrobial use frequency (qualitative data) from specific studies suggests a diversity of antimicrobials used for both prophylactic and therapeutic purposes, as well as growth promotion, although results are difficult to compare across studies. Tetracyclines, fluoroquinolones and beta-lactams/aminoglycosides were the common antimicrobials used on the farms regardless of the species of food animal in the various studies. This probably suggests that these antimicrobials are readily available in these countries over the counter and are inexpensive compared to third-generation antimicrobials. This finding concurs with observations by Chantziaras et al. (50), in one of their studies on antimicrobial use in livestock production in Europe. This could be due to the non-existent/lack of enforcement of regulatory measures in developing countries which has resulted in abuse of those classes of antimicrobials in food production animals unlike in developed countries where it can be attributed to the prescription tendency of veterinarians. The unregulated use of critically important antibiotics like fluoroquinolones used in human medicine in food producing animals is worrisome (51).

### Antimicrobial and multidrug resistance

The resistance prevalence ranged from 6.5 to 100% and that of multidrug resistance from 33.3 to 100%. This could be due to unregulated use and administration of antimicrobials which exert selection pressure on the emergence of resistant bacterial strains. Secondly, the numerous resistance patterns also imply that livestock practices in Africa are reliant on antimicrobials (52). Regarding the species type, poultry had the highest prevalence of resistant or multidrug resistant *Escherichia coli* in our study. This can be exemplified by rapid growth and high financial returns and easy management by farmers in close proximity (Intensive system) where antimicrobial usage is high to curb morbidity and mortality. Our findings coincided with studies carried out in developing countries like Thailand and Vietnam (53, 54) but higher than in developed countries like Denmark which was in the range: (of 4–65%) (55–58). This is probably because of long term monitoring and surveillance, biosecurity measures, and the ban of growth promoters in food producing animals in developed countries. Such policies and measures would have an impact on the emergence, development and spread of antimicrobial resistance in food animal production in Sub-Saharan Africa.

The pathogen prevalence in poultry and cattle and the level of antimicrobial resistance and susceptibility test to different antimicrobials is enough evidence to guide antimicrobial selection and support for judicious use. However, the lack of antimicrobial use monitoring systems and research capacity limitations typical of many LMICs represent other challenges (8). Therefore, animal health workers or veterinarians rarely collect samples for bacterial identification and antimicrobial sensitivity tests. Our findings demonstrate that antimicrobial resistance in food producing animals is a problem and is associated with the unregulated administration of antimicrobials by farmers and also the non-existence of regulatory use measures. Bearing in mind that antimicrobial resistance is of worldwide concern in humans and livestock, policies based on regulatory control of antimicrobial use are necessary and farmers training on judicious antimicrobial use to reduce the risk/number of AMR pathogens transmitted to humans *via* direct and indirect contact with livestock and poultry.

This review has some limitations. We managed to gain full access to two online databases and so there was a possibility of not recovering key articles due to search strategy boundaries as well as search interfaces. However, we minimized this effect by referring to the reference list of significant research articles. This review covers 24 articles published in English so there is a possibility that there were similar articles published in other languages in some Sub-Saharan countries which may offer similar or different findings. Questionnaire based antimicrobial use surveys cannot detect misuse and off-label use, and as such approaches like prescription, reviews are needed. Furthermore, we included a few developing countries mostly those from Southeast Asia because those countries in addition to increased levels of animal product production and consumption where AMU is inevitable to meet the demand of the increased population are also considered to be a hotspot of infectious disease and AMR. Future studies should compare developing countries not included in this study to those developed countries not considered in terms of AMU and AMR.

## Conclusion

This study has revealed a high level of antimicrobial usage, especially tetracyclines, fluoroquinolones and beta-lactams/aminoglycosides in cattle and poultry production in sub-Saharan Africa. This is likely to intensify the already high prevalence of antimicrobial resistance and multidrug resistance in the region. This, coupled with low enforcement of antimicrobial regulatory measures in most of the sub-Saharan African countries is of concern to food animals and public health. Secondly, the review has indicated a deficit of studies on the estimates of quantity and quality of antimicrobials used in food producing animals (poultry and cattle) in sub-Saharan Africa yet they play a role in the overall picture of antimicrobial resistance This therefore has given us a node of focus for future research. The study has also confirmed that antimicrobials of veterinary importance as defined in the WHO list (59) as the highest priority critically important antimicrobials in humans were still used in poultry and cattle production.

## Data availability statement

The original contributions presented in the study are included in the article/Supplementary material, further inquiries can be directed to the corresponding author.

## Author contributions

RA conceived the idea, performed a literature search, data extraction, and drafted the first version of the manuscript. FD cleaned the data and revised the first version of the manuscript. MS, MM, and SK were involved in the revision of the final manuscript before submission. All authors contributed to the article and approved the submitted version.

## Conflict of interest

The authors declare that the research was conducted in the absence of any commercial or financial relationships that could be construed as a potential conflict of interest.

## Publisher's note

All claims expressed in this article are solely those of the authors and do not necessarily represent those of their affiliated organizations, or those of the publisher, the editors and the reviewers. Any product that may be evaluated in this article, or claim that may be made by its manufacturer, is not guaranteed or endorsed by the publisher.
