# Supplementary material for: Antimicrobial use in cattle and poultry production on occurrence of multidrug resistant Escherichia coli. A systematic review with focus on sub-Saharan Africa
Source: Front Vet Sci. 2022 Oct 24;9:1000457. doi: 10.3389/fvets.2022.1000457 (PMC9637661; doi:10.3389/fvets.2022.1000457)
Supplement: Supplementary file 1 [file Data_Sheet_1.docx]

**Supplementary Table 1:** Search strategy

Search string used with resulting numbers of citations, 2008-2018

| **Search string** | **Numbers of articles retrieved per Library** |
| --- | --- |
|  |  |
| **PubMed search:** Antimicrobial usage) OR Consumption) OR Quantity AND Quality AND Livestock) OR Poultry OR Chicken OR Cattle OR Dairy OR Beef AND Sub-Saharan Africa OR Djibouti OR Comoros OR Madagascar OR Malawi OR Seychelles OR Cameroon OR Central African Republic OR Chad OR Congo OR Congo, Dem. Rep OR Equatorial Guinea OR Gabon OR South Sudan OR Sudan OR Botswana OR Lesotho OR Benin OR Burkina Faso OR Ghana OR Guinea OR Guinea-Bissau OR Mauritania OR Niger OR Senegal OR Sierra Leone OR Togo OR Burundi OR Ethiopia OR Kenya OR Mozambique OR Rwanda OR Somalia OR Tanzania OR Uganda OR Zambia OR Zimbabwe OR Angola OR Namibia OR South Africa OR Gambia OR Liberia OR Ivory coast OR Mali OR Nigeria AND 2008 to 2018 | 505 |
| Reference list of significant papers | 12  (35 duplicates) |
| **Total** | **482 citations** |
| **Google Scholar search:** Antimicrobial usage OR Consumption OR Quantity AND Quality AND Livestock OR Poultry OR Chicken OR Cattle OR Dairy OR Beef AND Sub-Saharan Africa followed by individual countries | 996 |
| Reference list of significant papers | 14  (46 duplicates) |
| **Total** | **964 citations** |

**Supplementary Table 2:** Characteristics of eligible studies included

| **Country** | **Food animal** | **Sample type** | **Study site** | **% AMU** | **Resistance determined** | **Grade** | **Reference** |
| --- | --- | --- | --- | --- | --- | --- | --- |
|  |  |  |  |  | R MDR |  |  |
| Nigeria | Poultry | n/a | Farm | 100 | n/a n/a | 4 | Adebowale, Adeyemo, Awoyomi, Dada & Adebowale, 2016 |
| Nigeria | Poultry | F/droppings | Farm | 100 | + n/a | 4 | Awogbemi, Adeyeye & Akinkunmi,2018 |
| Uganda | Poultry | n/a | Farm | 96.7 | n/a n/a | 3 | Bashahun & Odoch, 2015 |
| Ghana | Poultry | n/a | Farm | 98 | n/a n/a | 4 | Boamah, Agyare, Odoi &  Dalsgaard, 2016 |
| Cameroon | Poultry | n/a | Farm | 100 | n/a n/a | 4 | Kamini, Keutchatang, Mafo, Kansci &Nama, 2016 |
| Nigeria | Poultry | n/a | Farm/drug shop | 100 | n/a n/a | 3 | Geidam et al.,2012 |
| Nigeria | Poultry | n/a | Farm | 100 | n/a n/a | 4 | Oluwasile, Agbaje, Ojo &  Dipeolu, 2014 |
| Nigeria | Cattle/sheep/goats | n/a | Pastoralists | 100 | n/a n/a | 4 | Alhaji & Isola, 2018 |
| Nigeria | Cattle/Goats/Sheep | n/a | Drug shops | ---- | n/a n/a | 4 | Adesokan, Akanbi, Akanbi & Obaweda, 2015 |
| Nigeria | Poultry/Goats/Sheep | F/droppings | HH | 100 | + + | 4 | Okpara et al.,2018 |
| Sudan | Poultry/Cattle/Goats | n/a | Farm/HH | 95 | n/a n/a | 4 | Eltayb, Barakat, Marrone,  Shaddad & Lundborg, 2012 |
| South Africa | Poultry/Cattle/Goats/ Sheep | n/a | Pharmaceuticals/ Drug shops | ---- | n/a n/a | 4 | Eagar, Swan, &Van Vuuren, 2012 |
| Nigeria | Poultry &Pigs | n/a | Farms | 67 | n/a n/a | 4 | Amaechi, 2014 |
| Zambia | Cattle | Faecal | Farm/drug shops | ---- | + n/s | 4 | Mainda et al., 2015 |
| Tanzania | Cattle/Goats/Sheep | n/a | Pastoral herds | 74 | n/a n/a | 4 | Caudell et al., 2017 |
| Uganda | Cattle/Pigs/goats/ Chicken | Faecal | Farms | 100 | + + | 4 | Okubo et al., 2018 |
| Ghana | Cattle /Goats/pigs/ Chicken &Sheep | Faecal | Farms | 98 | + + | 3 | Donkor, Newman & Yeboah-Manu, 2012 |
| Ethiopia | Cattle/ Poultry | n/a | Farms | 80 | n/a n/a | 4 | Tufa et al., 2018 |
| Zambia | Cattle | Faecal | Pastoral herds | 100 | + n/s | 4 | Mubita et al., 2008 |
| Sudan | Poultry | n/a | Farms | 93 | n/a n/a | 3 | Sirdar, Picard, Bisschop & Gummow, 2012 |
| Cameroon | Poultry | Chicken tissues | Farms | 80 | + n/s | 4 | Guetiya et al.,2016 |
| Cameroon | Cattle | Penicillin & tetracycline | Pastoral herds | 69 | n/a n/a | 4 | Vougat Ngom et al, 2017 |
| Tanzania | Poultry | Chicken liver | HH/Farms | 100 | n/a n/a | 4 | Nonga, Mariki, Karimuribo & Mdegela, 2009 |
| Nigeria | Cattle/poultry/sheep  Goats/Rabbits | n/a | Farms | 99.1 | n/a n/a | 4 | Olufemi, Eniola, Ademola & Morenike, 2015 |

AMU, Antimicrobial use; F, Faecal; HH, House Hold; MDR, Multidrug resistance; n/a, not applicable; n/s not specified; +, positive; R, Resistance
